# Spatial and temporal mapping of early alphaherpesvirus invasion routes into the mouse central nervous system

**DOI:** 10.1007/s13365-025-01278-3

**Published:** 2025-09-28

**Authors:** Viktoria Korff, Issam El-Debs, Julia Sehl-Ewert

**Affiliations:** Department of Experimental Animal Facilities and Biorisk Management, Friedrich‐Loeffler‐Institut, Südufer 10, 17493 Greifswald-Insel Riems, Germany

**Keywords:** Pseudorabies virus, Mouse model, Neuroinvasion, Trigeminal route, Olfactory route, Spatial mapping

## Abstract

Alphaherpesviruses such as Herpes Simplex Virus 1 (HSV-1) and Pseudorabies virus (PrV) invade the central nervous system (CNS) via peripheral nerves. While olfactory and trigeminal pathways are well-known, additional cranial routes remain underexplored. Using a PrV-∆UL21gfp/US3∆kin mutant in CD1 mice, we mapped early neuroinvasion (4–96 hpi) by immunofluorescence and RNA in situ hybridization. Viral antigen and lytic viral gene expression (UL19 RNA) were detected in the olfactory epithelium, vomeronasal organ, incisors, palate, olfactory bulb, and brainstem. These results indicate multineural CNS access involving olfactory (I), trigeminal (V), glossopharyngeal (IX), and hypoglossal (XII) nerves, highlighting this model’s value for studying early alphaherpesvirus spread.

**Supplementary Information:**

The online version contains supplementary material available at 10.1007/s13365-025-01278-3.

## Introduction

Alphaherpesviruses such as Herpes Simplex Virus 1 (HSV-1) and Pseudorabies Virus (PrV), are neurotropic, double-stranded DNA viruses. Following productive infection of the oral and nasal mucosa, viral particles enter peripheral sensory neurons (Spear et al 2000; Mettenleiter 2003) and travel retrogradely to the trigeminal ganglion (TG) and other peripheral sensory and autonomic ganglia, where they establish life-long latency (Smith 2012; Koyuncu et al 2018; Hill et al 2001, 1975; Duarte et al 2019). From there, they may reactivate followed by anterograde movement towards the periphery or continue to spread retrogradely, occasionally reaching the central nervous system (CNS) where they can establish life-threatening inflammation of the brain (Sivasubramanian et al 2022; Yao et al 2014).

Despite extensive research, the precise pathways of alphaherpesviral neuroinvasion remain incompletely defined. Two main invasion pathways to the CNS have been implicated: the trigeminal and olfactory nerve (Held and Derfuss 2011; Shivkumar et al 2013).

The trigeminal nerve provides direct monosynaptic input to brainstem nuclei such as the principal sensory (Pr5) and spinal trigeminal (Sp5) nuclei, which in turn project to higher brain regions (Ezure et al 2001; Arbuthnot et al 1990). Alternatively, the olfactory nerve, composed of axons from olfactory sensory neurons in the olfactory epithelium (OE), terminates in the olfactory bulb (OB), which connects to mesiotemporal areas including the piriform and entorhinal cortices (Menendez and Carr 2017; Esiri 1982; Twomey et al 1979; Niemeyer et al 2024; Sosulski et al 2011; Barrios et al 2014).

Beyond these classical routes, recent studies suggest that additional cranial nerves—including the glossopharyngeal (IX), vagus (X), and hypoglossal (XII) nerves—may contribute to CNS entry. HSV-1 has been detected in nervus vagus-associated brainstem nuclei (Niemeyer et al 2024). Furthermore, varicella-zoster virus, another alphaherpesvirus, has been shown to infect the vagus nerve (Chen et al 2011; Gershon et al 2015) supporting a model of multineural entry.

Once in the CNS, alphaherpesviruses can cause severe neurological disease (Stahl and Mailles 2019). HSV-1 is the primary cause of sporadic herpes simplex encephalitis (HSE) in humans (Bradshaw and Venkatesan 2016) with a tropism for mesiotemporal structures. PrV, a closely related virus, induces fatal encephalitis in non-suid species, including mice, which are highly susceptible (Mettenleiter 2000; Babic et al 1994).

A specific PrV mutant, PrV-ΔUL21/US3Δkin—lacking the tegument protein pUL21 and encoding a kinase-deficient pUS3—exhibits pronounced neurotropism in CD1 mice, with early involvement of brainstem nuclei followed by the establishment of productive infection in mesiotemporal regions. Despite the characteristic pattern resembling HSE, the animals typically survive the infection although clinical signs may occur (Sehl et al 2020). This model provides a robust system for investigating alphaherpesviral neuroinvasion.

In this study, we used spatial–temporal mapping techniques to investigate early CNS entry routes of the gfp-tagged PrV mutant PrV-∆UL21gfp/US3∆kin in mice following intranasal inoculation. By combining immunofluorescence and RNA in situ hybridization at defined time points, we identified viral spread through olfactory, trigeminal, and additional cranial nerve pathways, providing new insights into the mechanisms of alphaherpesviral CNS invasion.

## Material and methods

### Virus

PrV strain PrV-∆UL21gfp/US3∆kin was kindly provided by B. G. Klupp, Friedrich-Loeffler-Institut Greifswald-Insel Riems. As previously described (Sehl et al 2020, Korff et al 2025), PrV-∆UL21gfp/US3∆kin was derived from the PrV wildtype strain Kaplan (Kaplan and Vatter 1959). The virus was propagated in rabbit kidney (RK13) cells and cultured at 37 °C in minimum essential medium (MEM) with 10% fetal calf serum (FCS) (Invitrogen).

### Animal experiments

We used six to eight-week-old female CD1 mice (Charles River Laboratory), as this strain serves as the standard infection model in our laboratory (Sehl et al 2020). The animals were housed in groups of four in conventional cages (type II L) under Biosafety Level 2 (BSL 2) conditions at the animal facility of the Friedrich-Loeffler-Institut, Greifswald-Insel Riems. Environmental conditions were kept constant, with a 12-h light–dark cycle (daylight intensity: 60%) and a temperature range of 20–24 °C. Mice had ad libitum access to a standardized diet (ssniff Ratte/Maus-Haltung) and fresh drinking water. To promote animal welfare, bedding (ssniff Spezialdiäten Abedd Espen CLASSIC), nesting material (PLEXX sizzle nest), and environmental enrichment (PLEXX Aspen Bricks medium, mouse smart home, mouse tunnel) were provided.

After a one-week acclimatization period, mice were deeply anesthetized by intraperitoneal injection of 200 µl of a ketamine-xylazine mixture (ketamine: 60 mg/kg; xylazine: 3 mg/kg) diluted in 0.9% sodium chloride. Following the onset of anesthesia, each nostril was inoculated with 5 µl of PrV-∆UL21/US3∆kin suspension in cell culture medium, resulting in a total inoculation dose of 1 × 10^4^ PFU/ml.

A total of nine groups of mice (n = 4 per group) were included in the study, with scheduled euthanasia at defined time points post-infection: 4, 8, 16, 24, 36, 48, 60, 72, and 96 h post-infection (hpi). Animals were continuously monitored (24/7) throughout the experimental period. Clinical evaluation was based on a predefined scoring system (Sehl et al 2020) encompassing three categories: (I) external appearance, (II) behavior and activity, and (III) body weight. Each category was scored from 0 to 3. The humane endpoint for euthanasia was defined as either a score of 3 in one category or a score of 2 across all three categories.

Animals were euthanized under deep isoflurane anesthesia, preceded by Carprofen (Rimadyl, Pfizer, 10 mg/kg, subcutaneous) analgesia. Under deep anesthesia, the right atrium was incised, and a 26-gauge needle was inserted into the left ventricle to facilitate perfusion with phosphate-buffered saline (PBS), followed by fixation with 4% paraformaldehyde (PFA), as described by (Gage et al 2012). After perfusion, euthanasia was completed by decapitation, with the head removed at the level of the first cervical vertebra. Heads were fixed in 4% PFA for at least one week and then decalcified for seven days in Osteosoft ® (Merck).

### Detection of viral antigen – immunolabeling

**Fig. 1:**
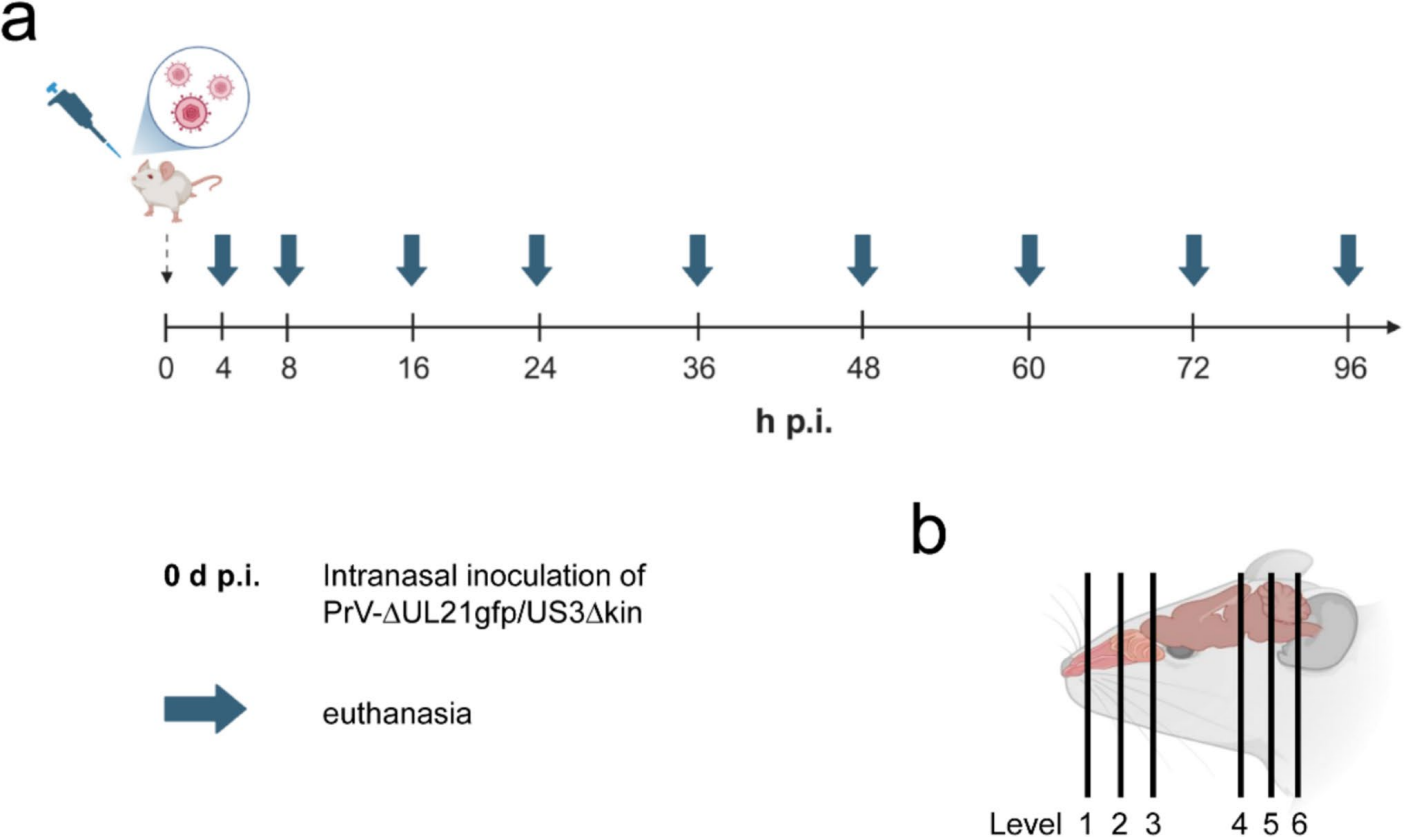
(**a**) Sequential timeline of necropsies and tissue collection. Six- to eight-week-old CD1 mice were intranasally inoculated with PrV-∆UL21gfp/US3∆kin (0 dpi) and sacrificed at the indicated time points. (**b**) Schematic illustration of analyzed head regions. Serial 50 µm coronal sections were collected at six defined rostrocaudal levels: L1 (posterior upper incisors), L2 (incisive papilla), L3 (second palatine crest/olfactory bulb), L4 (midbrain), L5 (cerebellum/pons), and L6 (medulla), for detailed analysis. Created in BioRender. Korff, V. (2025) https://BioRender.com/y01iyic

Three fixed and decalcified heads per time point were trimmed into six sections and cut into 50 µm thick coronal slices using a cryostat (CryoStar™ NX70, Epredia). Tissue sections were collected at defined anatomical landmarks following established protocols (Rao et al 2011; OECD 2010) including the posterior part of upper incisors (L1), incisive papilla (L2), second palatine crest/olfactory bulb (L3), midbrain (L4), cerebellum/pons (L5) and medulla (L6) (Fig. 1b). Tissue slices were transferred onto Super-Frost-Plus slides (Carl Roth GmbH, Germany) in a frozen state, air-dried at room-temperature (RT) for 5–10 min, and stored in PBS at 4 °C until further processing.

Prior to immunolabeling, slices were circumscribed using a grease pencil (Vector Laboratories, H-4000) to ensure precise application of reagents. In a humified chamber, tissue samples were permeabilized for 10 min using 0.25% Triton X-100 and 0.2% gelatin in PBS (permeabilization buffer). Blocking was performed with 5% bovine serum albumin (BSA, Merck, A7979) in permeabilization buffer for 1 h at RT.

For primary antibody staining, samples were incubated for 1 h at RT with rabbit polyclonal anti-PrV glycoprotein B (gB) antibody (1:2000, (Kopp et al 2003)) and chicken polyclonal anti-neurofilament protein antibody (NEFM, 1:2000, ThermoFisher, PA1-16758), both diluted in 0.1% BSA in permeabilization buffer. After washing (twice for 2 min in PBS, followed by 10 min in permeabilization buffer), secondary antibodies were applied: goat anti-rabbit Alexa Fluor 488 (1:1000, Invitrogen, AB150077) and goat anti-chicken Alexa Fluor 568 (1:1000, Invitrogen, A11041). Incubation was performed for 1 h at RT under light-protected conditions.

Following secondary antibody incubation, samples were washed twice for 2 min in PBS and counterstained with Hoechst 33258 (1:20,000, Sigma-Aldrich, 94403) for 5 min at RT, protected from light. After two final washes in PBS (2 min each), samples were incubated for 10 min at RT in a light-protected 10 mM copper (II) sulfate (CuSO_4_)/50 mM ammonium chloride (NH_4_Cl) solution (wash buffer 2) to reduce background fluorescence. Finally, slices were rinsed in deionized water for 2 min, mounted with one drop of mounting medium (ibidi), and air-dried for 24 h.

Fluorescently labeled sections were analyzed with a THUNDER Imaging system (THUNDER Imager Tissue; Leica, Germany). Images were processed with arivis Vision4D (ZEISS).

### In-Situ hybridization/RNA Scope™

One head per time point (24, 60, 72hpi) was trimmed into 6 sections as described in the section immunofluorescence (Fig. 1b), embedded in paraffin wax and cut at 3 µm thick slices using a rotating microtome (Hyrax M55, Zeiss). Sections were mounted on Super-Frost-Plus-Slides (Carl Roth GmbH, Germany) and dried for 2 h at 40 °C, and were stored at RT until staining.

To detect PrV mRNA, RNAscope™ in situ hybridization (Wang et al 2012) was performed on the formalin-fixed, paraffin-embedded (FFPE) head sections following the protocol for the RNAscope™ 2.5 HD Reagent Kit-RED (ACD Inc., Cat. No. 322350). A C1 probe was designed to target lytic viral gene expression with PrV UL19 mRNA (probe: V-SHSV-UL19, specific to the 66973–68498 bp region of UL19; ACD Inc., Cat. No. 548251). Signal detection was based a chromogenic alkaline phosphatase (AP) reaction, producing red-colored punctate signals for each RNA transcript. As internal control, the murine housekeeping gene Mm-Ppib (ACD Inc., Cat. No. 313911, C1), encoding peptidyl-prolyl cis–trans isomerase B, was used. The negative control consisted of the Escherichia coli DapB transcript (RNAscope™ Negative Control Probe; ACD Inc., Cat. No. 310043) (S1 Fig.).

Before hybridization, slides were baked at 60 °C for 1 h deparaffinized in xylene (2 × 5 min) and rehydrated in 100% ethanol (2 × 1 min) at RT. After air-drying, sections were treated with hydrogen peroxide (H_2_O_2_; ACD Inc., Cat. No. 322330) for 10 min at RT. Target retrieval was carried out in boiling antigen retrieval buffer (ACD Inc., Cat. No. 322000) using a pressure cooker (Sichler, Germany, NX-3213–675) for 15 min, followed by washing in distilled water and dehydration in 100% ethanol. After air-drying, sections were circumscribed with a grease pencil (Vector Laboratories, H-4000) to confine reagent application to the tissue area. Slides were then treated with Protease Plus (ACD. Inc, Cat. No.322330) for 15 min at 40 °C in the ACD HybEZ™ II Hybridization System (ACD Inc., Cat. No. 321711) (HybEZ oven). Slides were rinsed in distilled water and incubated with the specific probe and controls for 2 h at 40 °C in the HybEZ oven, followed by two washes (2 min each) in the wash buffer provided (ACD Inc., Cat. No. 310091). Preamplification was performed by applying Amp 1 for 30 min at 40 °C. Amplification was continued with Amp 2 (15 min) and Amp 3 (30 min), both at 40 °C. These steps enable signal enhancement by sequential binding of amplification complexes. Chromogenic signal development for probe C1 was performed by incubating slides with Amp 4 enzyme solution for 15 min at 40 °C in the HybEZ oven, followed by chromogenic substrate Amp 5 for 30 min at RT, and the AP blocker Amp 6 for 15 min at RT. For visualization of the red signal, slides were treated with a 1:60 mixture of Red-B to Red-A for 10 min at RT. After signal detection, slides were then washed twice (2 min each) in wash buffer. All amplification and signal development reagents were included in the RNAscope™ 2.5 HD Reagent Kit-RED (ACD. Inc, Cat. No. 322350). Slides were counterstained with Mayer`s Hematoxylin staining solution for 30 s at RT and rinsed 5 min in tap water for bluing. After drying on a 60 °C heat plate (MEDITE, BD00575) for 15–30 min and cooling for 5 min, slides were immersed in fresh xylene for 5 min and mounted using EcoMount (Biocare Medical, BRR897L).

All slides were analyzed using a Zeiss Axio Scope.A1 microscope equipped with 5x, 10x, 20x, and 40 × N-ACHROPLAN objectives (Carl Zeiss Microscopy GmbH, Jena, Germany) and scanned with the NanoZoomer (Hamamatsu, S60) scanner.

## Results

The primary objective of this study was to define cranial nerve-mediated entry of the PrV-ΔUL21gfp/US3Δkin mutant into the CNS using a standardized mouse intranasal infection model.

To achieve fine-scale spatial–temporal mapping of viral neuroinvasion, intranasally PrV-∆UL21gfp/US3∆kin inoculated animals were sacrificed at defined time points post-infection (4, 8, 16, 24, 36, 48, 60, 72, and 96 hpi) and processed for subsequent histological and molecular analyses (Fig. 1a).

### Clinical evaluation

No clinical signs were observed in any of the mice throughout the experimental period.

### Mapping viral entry routes into the CNS

#### Spatial and temporal distribution of viral antigen

**Fig. 2:**
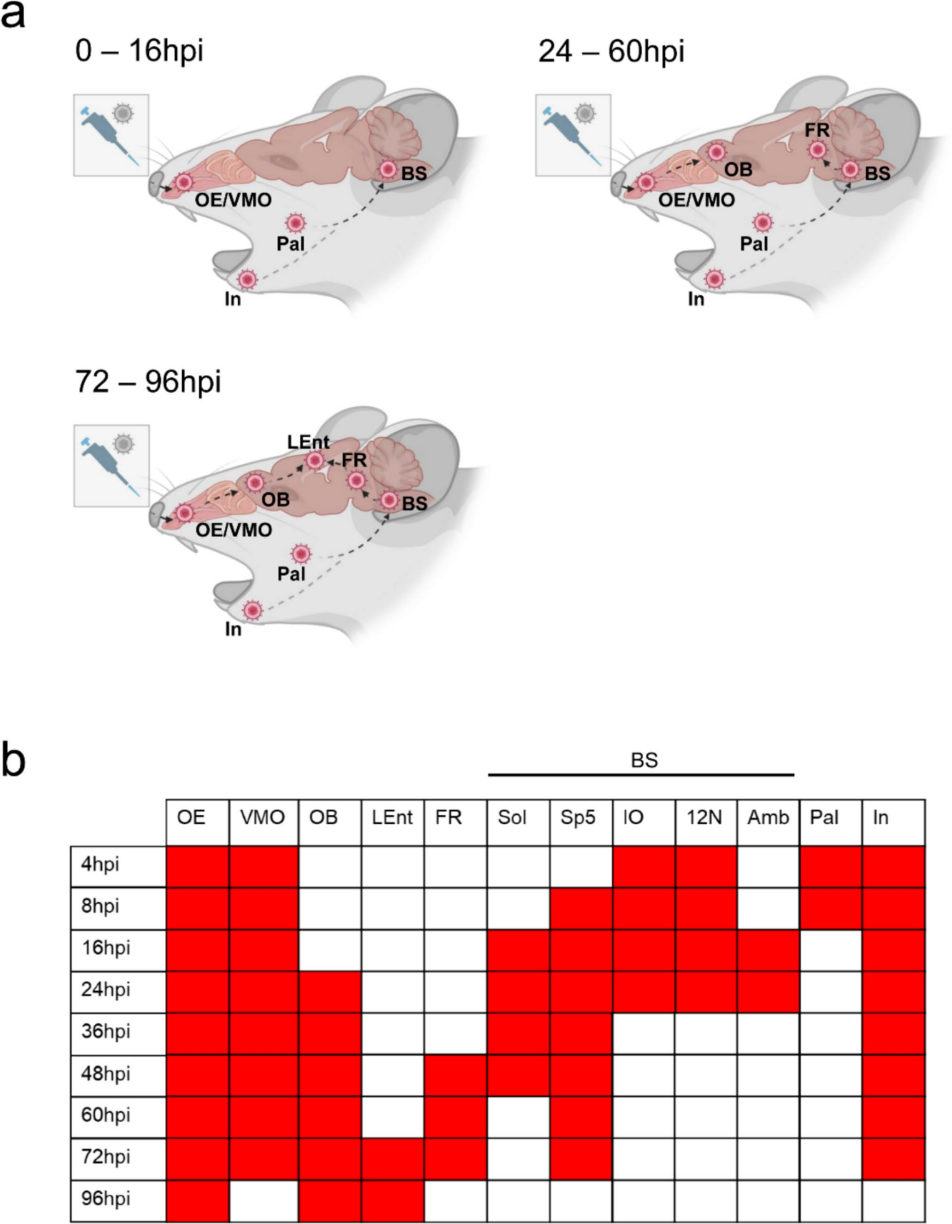
Viral antigen distribution in the murine head following intranasal inoculation with PrV-∆UL21gfp/US3∆kin at different time points. (**a**) Viral antigen was detected in the olfactory epithelium (OE), vomeronasal organ (VMO), palate (Pal), incisor region (In) and brainstem (BS) at early time points (0–16 hpi), followed by signal in the olfactory bulb (OB) and reticular formation (FR) (24–60 hpi), and later in the lateral entorhinal cortex (LEnt) (72–96 hpi). The spatiotemporal distribution of viral antigen was visualized by immunofluorescence and is schematically indicated by arrows. Created in BioRender. Korff, V. (2025) https://BioRender.com/jtrk9ey (**b**) Schematic overview of viral antigen localization in brain regions of infected mice (*n* = 3) at different time points. Colored boxes (red) indicate the presence of viral antigen in the corresponding anatomical regions in at least 2 mice. Sol = nucleus of the solitary tract, Sp5 = spinal trigeminal nucleus, IO = inferior olive, 12 N = hypoglossal nucleus, Amb = nucleus ambiguus

**Fig. 3:**
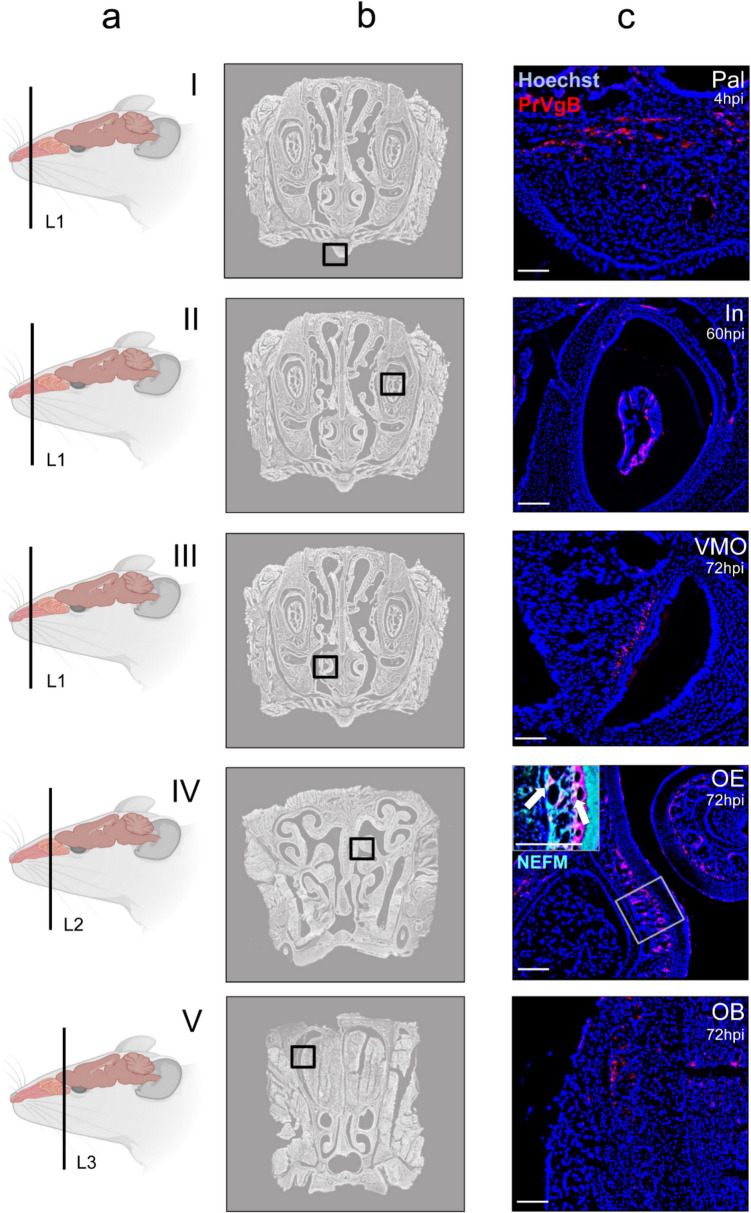
Immunofluorescence analysis of viral antigen distribution in the CD1 mouse head following intranasal inoculation with PrV-∆UL21gfp/US3∆kin. (**a**) Schematic illustration of coronal sections through the murine head (L1-3). Created in BioRender. Korff, V. (2025) https://BioRender.com/fjixhs4 (**b**) Overview of the anatomical regions analyzed in the coronal sections. (**c**) Representative immunofluorescence images showing PrV glycoprotein B (gB) expression in cranial sections from mice infected with PrV-∆UL21gfp/US3∆kin. Immunolabeling was performed using a rabbit polyclonal anti-PrV gB serum and a goat anti-rabbit Alexa Fluor 488-conjugated secondary antibody (visualized in red). Neurofilament protein was labeled using a chicken polyclonal anti-NEFM antibody and a goat anti-chicken Alexa Fluor 568-conjugated secondary antibody (cyan). Double-labeling of NEFM and PrVgB is indicated by arrows. Cell nuclei were counterstained with Hoechst (blue). In = incisor, Pal = palate, VMO = vomeronasal organ, OE = olfactory epithelium, OB = olfactory bulb. Scale bar 100 µm

**Fig. 4:**
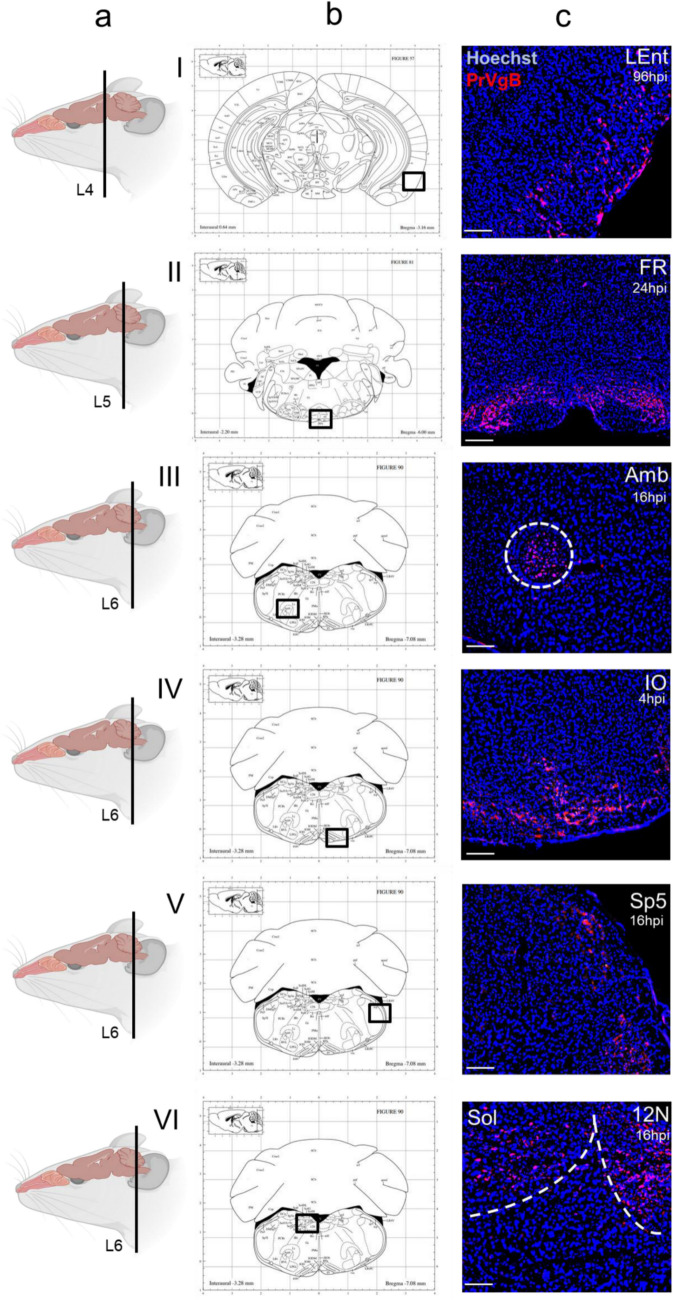
Immunofluorescence analysis of viral antigen distribution in the murine brain following intranasal inoculation with PrV-∆UL21gfp/US3∆kin. (**a**) Schematic illustration of coronal sections through the murine head (L4-6). Created in BioRender. Korff, V. (2025) https://BioRender.com/fjixhs4 (**b**) Schematic representation of the stained brain regions, shown in accordance with The Mouse Brain in Stereotaxic Coordinates (Paxinos and Franklin, 2001). (**c**) Representative immunofluorescence images showing PrV glycoprotein B (gB) expression in coronal brain sections from PrV-∆UL21gfp/US3∆kin-infected CD1 mice. Immunolabeling was performed using a rabbit polyclonal anti-PrV gB serum and a goat anti-rabbit Alexa Fluor 488-conjugated secondary antibody (red). Nuclei were counterstained with Hoechst (blue). Sol = nucleus of the solitary tract, Sp5 = spinal trigeminal nucleus, IO = inferior olive, 12 N = hypoglossal nucleus, Amb = nucleus ambiguus, FR = reticular formation, LEnt = lateral entorhinal cortex. Scale bar: 100 µm

To define spatial dynamics of PrV-∆UL21gfp/US3∆kin entry into the CNS, immunofluorescence analysis was conducted on six standardized coronal sections of the murine head, corresponding to defined anatomical landmarks: the posterior part of upper incisors (L1), incisive papilla (L2), second palatine crest/olfactory bulb (L3), midbrain (L4), cerebellum/pons (L5) and medulla (L6). These sections were analyzed at defined time points post-inoculation (p.i.) (Fig. 1b). An overview of early viral antigen distribution is provided in Fig. 2a with detailed regional data in Fig. 2b. Representative immunofluorescence images of viral antigen dissemination in nasal and brain regions are presented in Figs. 3 and 4, respectively.

As early as 4–16 hpi, viral antigen was detected in the palate (Pal), incisors (In), vomeronasal organ (VMO), OE, and different brainstem (BS) regions. The virus progressively spread to the OB and reticular formation (FR), and by 72 hpi reached the temporal lobe, particularly the lateral entorhinal cortex (LEnt), where signal persisted until the termination of the experiment (96 hpi) (Fig. 2).

Region-specific analysis revealed transient signals in the Pal (Fig. 3I, ≤ 8 hpi), while antigen remained detectable in the In (Fig. 3II) and VMO (Fig. 3III) until 72 hpi. In the OE (Fig. 3IV), persistent viral labeling was observed from 4 to 96 hpi and was confirmed by co-localization with the neurofilament marker NEFM trough double immunolabeling. OB involvement (Fig. 3V) started at 24 hpi and was detectable until 96 hpi. In the BS, early infection of the inferior olive (IO) and hypoglossal nucleus (12N) was noted from 4 to 24 hpi (Fig. 4IV, VI), followed by Sp5 (8–72 hpi; Fig. 4V), nucleus ambiguus (Amb; 16–24 hpi; Fig. 4III), and nucleus of the solitary tract (Sol; 16–48 hpi; Fig. 4VI). FR involvement (Fig. 4II) was evident from 48 to 72 hpi, while viral antigen appeared in the LEnt of the temporal lobe (Fig. 4I) from 72 hpi onward.

#### Spatial detection of viral mRNA

**Fig. 5:**
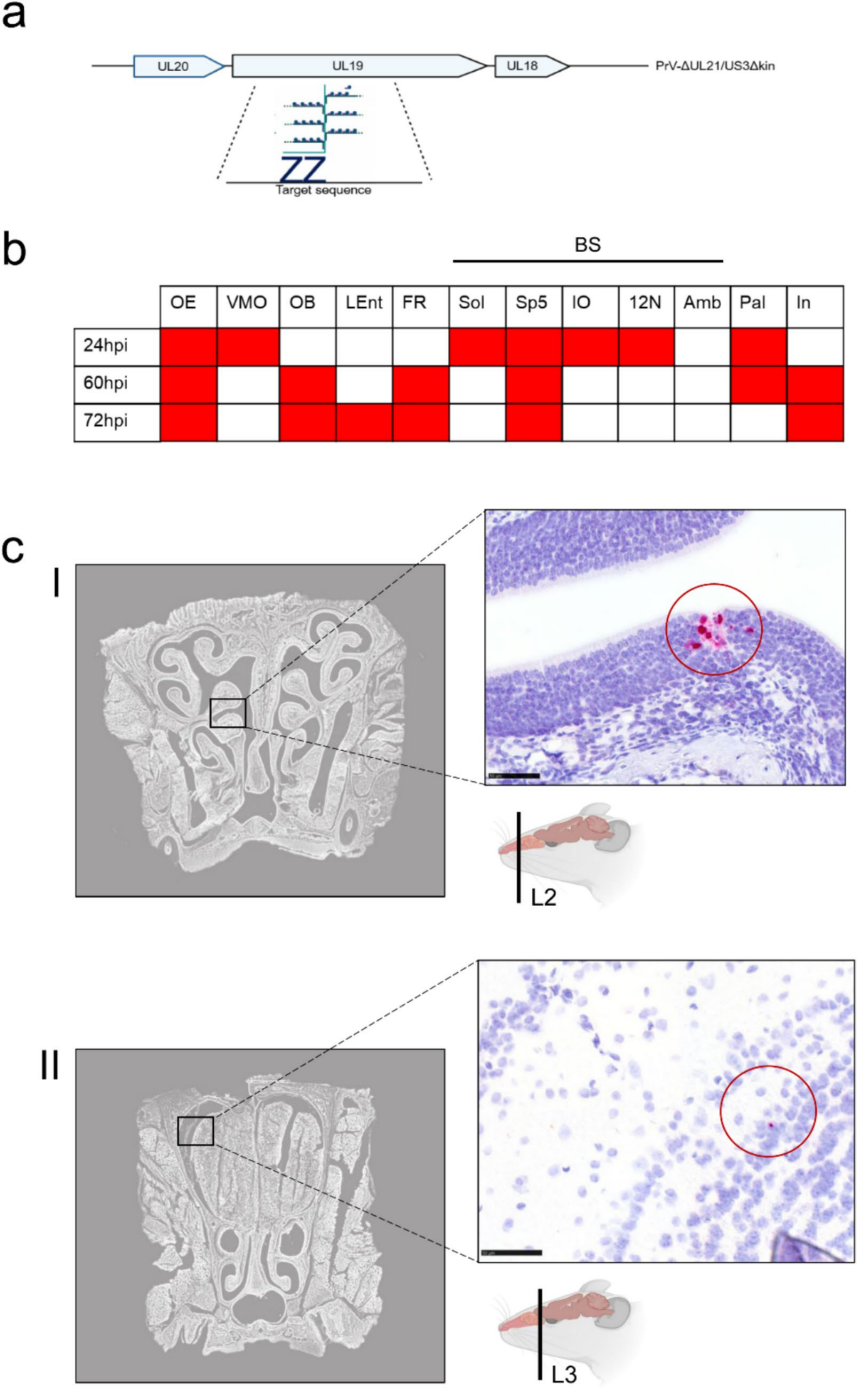
In situ hybridization-based detection of PrV UL19 RNA in the CD1 mouse head following intranasal inoculation with PrV-∆UL21gfp/US3∆kin. (a) Schematic representation of the UL19-specific probe design (V-SHSV-UL19). V-SHSV-UL19 targets the 66973–68498 bp region of UL19 which is represented by the blue dots and connecting lines. (b) Schematic overview of viral mRNA detection in brain regions of infected mice (n = 1) at different time points. Colored boxes (red) indicate the presence of viral mRNA in the corresponding anatomical regions. (c) In situ hybridization of PrV UL19 RNA in murine coronal nose and brain Sects. 72hpi. Evidence of lytic viral replication is shown by RNA Scope™ detection of UL19 transcripts (red) in infected tissues. (I) RNA Scope™ signal for UL19 RNA in the olfactory epithelium. (II) RNA Scope™ signal for UL19 RNA in the olfactory bulb. Scale bar = 50 µm. Created in BioRender. Korff, V. (2025) https://BioRender.com/dzegatr

To corroborate the distribution of viral antigen, in situ hybridization was performed using RNA Scope™ (Wang et al 2012) targeting the UL19 gene in one representative animal per time point (Fig. 5a). While in situ hybridization largely confirmed antigen distribution, notable temporal and spatial differences were observed: RNA signals in the VMO were transient and restricted to 24 hpi, while OB labeling appeared only from 60 hpi onward. Amb was no longer positive beyond 24 hpi, whereas the palate remained transcriptionally active up to 60 hpi despite declining antigen levels (Fig. 5b). Exemplary images of the OE (I) and OB (II) at 72hpi are shown in Fig. 5c.

## Discussion

In the present study, we investigated the alphaherpesvirus neuroinvasive entry and characterized early viral spread using a murine intranasal inoculation model. By combining fine-scaled temporal and spatial analyses of viral antigen and mRNA expression, we defined the initial stages of CNS invasion.

Our data confirmed the established roles of the trigeminal and olfactory pathways for viral entry. Moreover, we detected viral antigen in brainstem nuclei linked to the glossopharyngeal and hypoglossal nerves. These findings indicate that these cranial nerves provide additional routes for CNS access.

Throughout the study, mice remained clinically asymptomatic and survived the entire observation period, consistent with previous work using this model, where first mild clinical signs typically develop after 6 days post-infection (Sehl et al 2020).

Within the first 16 h post-infection, viral antigen was detected in the Pal, In, VMO, OE and brainstem nuclei: Sp5, Sol, Amb, IO and 12N. Later spread included the OB, FR, and by 72 hpi, the LEnt of the temporal lobe, a known target in herpesviral encephalitis (Esiri 1982; Taylor et al 2005; Yong et al 2021; Armien et al 2010, Korff et al 2025).

The present findings are consistent with recent reports of brainstem involvement following intranasal alphaherpesvirus infection; notably, however, those studies have been limited to analyses at later post-infection time points, in contrast to the current investigation, which encompasses earlier phases of infection (Sehl et al 2020; Niemeyer et al 2024; Klopfleisch et al 2006, 2004). Of note, infection of the OE following PrV infection has been so far solely detected in pigs (Verpoest et al 2017). However, we could show antigen signaling as early as 4 hpi in the murine OE alongside with a recent study showing HSV-1 signals 7 hpi (Niemeyer et al 2024).

Antigen detection findings were largely corroborated by RNA in situ hybridization; however, temporal and spatial discrepancies were noted. These likely reflect methodological differences: while immunohistochemistry detects accumulated viral proteins, in situ hybridization captures transcriptional activity. Notably, RNA analysis was performed on a single animal per time point and limited to one 3 µm section per anatomical level, in contrast to 50 µm sections used for antigen detection. This design—chosen to qualitatively confirm viral gene expression—offers only a limited snapshot rather than a comprehensive overview. Accordingly, the RNA-based data should be regarded as confirmatory and interpreted with caution in terms of spatial and temporal resolution.

**Fig. 6:**
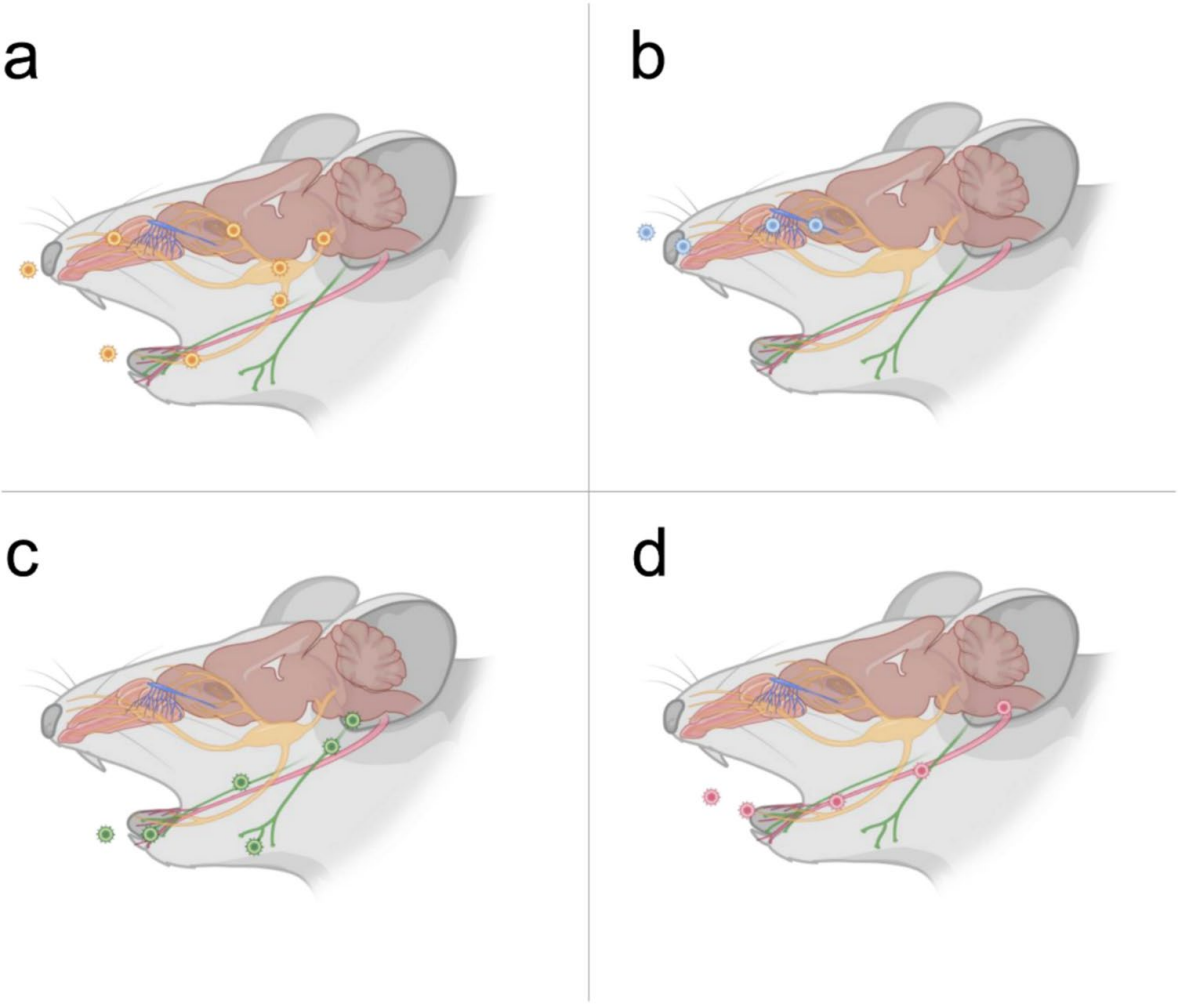
Schematic presentation of experimentally supported infection routes of alphaherpesviruses via cranial nerves (CN). (a) Trigeminal nerve (CN V) route (yellow). (b) Olfactory nerve (CN I) route (blue). (c) Glossopharyngeal nerve (CN IX) route (green). (d) Hypoglossal nerve (CN XII) route (red). Created in BioRender. Korff, V. (2025) https://BioRender.com/vggf7zl

Our findings suggest that PrV exploits multiple neuroinvasive routes to enter the CNS. The trigeminal pathway emerges as a predominant route (Held and Derfuss 2011; Sehl and Teifke 2020; Wang et al 2020), supported by viral antigen detection in Sp5 (Fig. 6a) (García-Guillén et al 2021; Landisman and Connors 2007) and functionally connected regions (Amb, IO, FR) (Isokawa-Akesson and Komisaruk 1987; van Ham and Yeo 1992; Panneton and Gan 2014). In addition, viral signals were observed in peripheral sites innervated by the maxillary, mandibular and ophthalmic branches of the trigeminal nerve, including the palate (Shankland 2001), nasal mucosa (Grunditz et al 1994; Yun et al 2024; Prendergast 2013) and incisivi (Fried and Gibbs 2014; Naftel et al 1999).

The olfactory pathway (Niemeyer et al 2024; Mori 2015; Steiner and Benninger 2013) is likewise confirmed by the presence of viral antigen in the OE, OB, and LEnt (Fig. 6b), which are anatomically and functionally interconnected, and provide access to limbic brain structures (Gretenkord et al 2019; Stenwall et al 2025). Detection of viral antigen in the VMO, as similarly reported by Mori et al 2005 for HSV-1, reinforces its role in CNS entry.

Evidence for glossopharyngeal nerve involvement (Fig. 6c) includes viral antigen in the Pal (Mu et al 2021) and brainstem nuclei Sol and Amb, which serve as the primary afferent target (Mizuno and Nomura 1986) and motor fiber origin (Standring 2016), respectively. Close anatomical and functional connectivity between these nuclei (Forstenpointner et al 2022; Stuesse and Fish 1984) strengthen the plausibility of glossopharyngeal-mediated CNS entry.

The hypoglossal nerve is implicated by detection of antigen in its origin site (Yamaguchi 2021), the hypoglossal nucleus (12N) (Fig. 6d), consistent with infection patterns observed in related alphaherpesviruses such as HSV-1 (Niemeyer et al 2024).

Considering that Sol receives dense vagal input (Chen and Liu 2025), and Amb contains vagal motor neurons (Isabella and Moens 2024), and that these nuclei are functionally linked to the glossopharyngeal nerve and integrated within the FR (Ruggiero et al 2000; Nasse et al 2008), it is reasonable to propose that the vagus nerve indirectly contributes to CNS invasion.

The dense anatomical and functional interconnections among brainstem nuclei innervated by different cranial nerves likely facilitate cross-communication and redistribution of viral particles between neuroinvasive pathways. For example, Sol is connected to both the trigeminal Sp5 (Okada et al 2019; Guan et al 1998) and 12 N (Guo et al 2020; Borke et al 1983). Further synaptic connections exist likewise between 12 N and Sp5 (Sousa Costa et al 2023; Streppel et al 2000) as well as between 12 N and FR (Holstege and Kuypers 1982; Streppel et al 2000; Borke et al 1983). Detection of viral antigen in the FR and associated nuclei involved in respiratory control and swallowing (Milsom et al 2004; Jean 2001) raises the possibility of an ingestion-related route of neuroinvasion. Although mice were intranasally inoculated, incidental ingestion of viral particles during application cannot be excluded. This may explain viral entry via glossopharyngeal and hypoglossal pathways, indicating that ingestion-associated mechanisms could complement direct neural invasion routes.

## Summary

This study identifies multiple early alphaherpesvirus neuroinvasion routes in a murine intranasal infection model (Fig. 6). The trigeminal and olfactory pathways are confirmed as primary entry points, alongside infection of the nasal mucosa. Furthermore, we demonstrate the involvement of the glossopharyngeal and hypoglossal nerves, showing that these cranial nerves also serve as important routes for CNS invasion. Given the extensive interconnectivity of brainstem nuclei associated with these nerves, including vagal inputs, the vagus nerve likely contributes as well. Viral spread into brainstem regions involved in respiratory and swallowing functions suggests a possible ingestion-associated entry route. These findings reveal a complex network of cranial nerve pathways facilitating early alphaherpesvirus neuroinvasion.

## Supplementary Information

Below is the link to the electronic supplementary material.

Supplementary file1 (PDF 125 KB)

## Data Availability

No datasets were generated or analysed during the current study.
