# Supplementary material for: Spatial and temporal mapping of early alphaherpesvirus invasion routes into the mouse central nervous system
Source: J Neurovirol. 2025 Sep 28;31(5):472–84. doi: 10.1007/s13365-025-01278-3 (PMC12618451; doi:10.1007/s13365-025-01278-3)
Supplement: Supplementary file 1 — Supplementary file1 (PDF 125 KB) [file 13365_2025_1278_MOESM1_ESM.pdf]

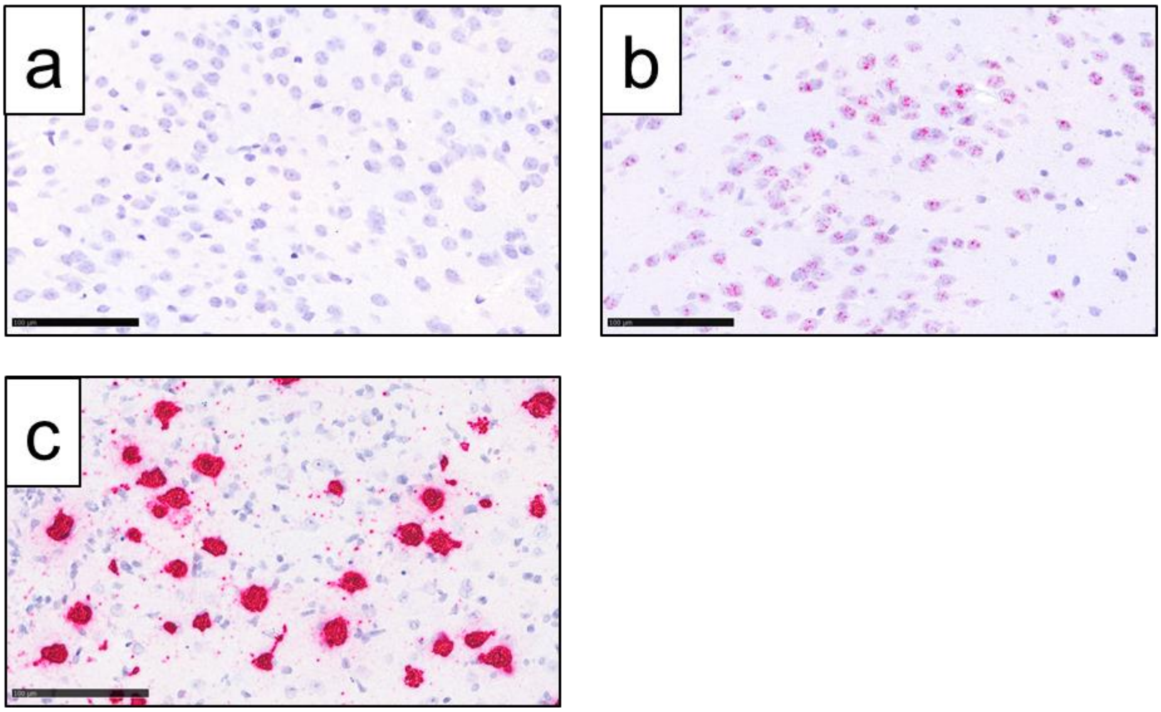

S1 Fig.: Representative images of RNA Scope™ control probe detection in the murine brain. (a) The Escherichia coli DapB probe served as a negative control probe and showed no specific signal. (b) The positive control probe Mm-Ppib (C1, red), demonstrated robust expression in neuronal tissue. (c) A brain section from a PrV-infected mouse at 14 days post-infection (dpi) was used as a positive control for UL19 probe detection during the acute phase of infection. Scale bar = 100 µm.
